# Missachtet und abgehängt: Kinder und Kinderrechte während der COVID-19-Pandemie

**DOI:** 10.1007/s12054-022-00466-3

**Published:** 2022-03-01

**Authors:** Jörg Maywald, Bianka Pergande

**Affiliations:** Fachhochschule Potsdam, Berlin, Deutschland

**Keywords:** Kinderrechte, Krise, COVID-19-Pandemie, Kindeswohl

## Abstract

Obwohl Kinder in besonderer Weise von den Einschränkungen aufgrund der COVID-19-Pandemie betroffen sind, wurden ihre Rechte in der Krise nur mit großer Verzögerung und sehr unvollständig berücksichtigt. Um die Interessen von Kindern während zukünftiger Krisen nicht erneut zu missachten, ist die Verwirklichung eines Kinderrechtsansatzes auf allen Ebenen notwendig. Besonders wichtig ist dafür die Verankerung der Kinderrechte im Grundgesetz.

Kinderrechte sind Menschenrechte, denen gerade in Zeiten der Krise eine überragende Bedeutung zukommt. Zwar ist anzuerkennen, dass bei Pandemien zum Schutz der öffentlichen Gesundheit internationale Menschenrechtsnormen bestimmte, zeitlich begrenzte Maßnahmen erfordern können, die die Wahrnehmung einiger Menschenrechte beschränken, um die Verbreitung eines Krankheitserregers einzudämmen. So kann es beispielsweise notwendig sein, das Recht von Kindern auf Versammlungsfreiheit oder ihr Recht auf Bildung vorübergehend einzuschränken.

Dies darf allerdings nur für die kürzeste angemessene Zeit und unter Wahrung der Verhältnismäßigkeit gelten. Zentrale kinderrechtliche Prinzipien wie der Vorrang des Kindeswohls und das Recht der Kinder auf Beteiligung müssen auch unter außergewöhnlichen Umständen beachtet werden.

## Umgang mit Kindern in der Corona-Zeit

Rückblickend können die rund zwei Jahre des Umgangs der Politik mit Kindern^1^ seit Ausbruch der Pandemie in Deutschland im März 2020 in sechs zeitliche Abschnitte aufgeteilt werden:

- Phase eins begann mit dem ersten Corona-Lockdown, der am 22. März 2020 in Kraft trat und nach sieben Wochen, am 4. Mai 2020, endete. Aufgrund der flächendeckenden Schließungen von Kitas, Schulen, Freizeiteinrichtungen und Spielplätzen waren Kinder in dieser Zeit vollständig auf ihre Familien zurückgeworfen. Da zu diesem Zeitpunkt noch wenig über Gefährlichkeit und Infektionswege des Corona-Virus bekannt war, kann eine solche zeitlich begrenzte gravierende Einschränkung von Kinderrechten als verhältnismäßig angesehen werden.
- Eine zweite Phase dauerte von Mai 2020 bis etwa zum Spätsommer desselben Jahres, dem Beginn des Kita- und Schuljahres 2020/2021. Dies war die Zeit von Beschränkungen der Rechte von bestimmten Gruppen von Kindern. Kinder, deren Eltern in so genannten systemrelevanten Berufen tätig waren, durften Kita und Schule besuchen, während die übrigen Kinder weiterhin ausgeschlossen waren. Mit dem Wohl von Kindern hatten diese Bestimmungen nichts zu tun, es ging ausschließlich darum, Kinder im Interesse einer Versorgung der Bevölkerung mit unentbehrlichen Gütern und Dienstleistungen „weg zu organisieren“. Kindgerechte Alternativen wären möglich gewesen und wurden an wenigen Orten auch praktiziert, zum Beispiel die Einführung von Schichtdiensten in den Bildungseinrichtungen, ergänzt durch Angebote der Notbetreuung.
- Phase drei setzte im Herbst 2020 ein und endete etwa auf dem Höhepunkt der zweiten Corona-Welle am Jahresende 2020. In dieser Zeit der Krise wurden Kinder erstmals ein wichtiges Thema der Politik. Es entstand die Befürchtung, dass viele Kinder aufgrund stark eingeschränkter Bildungsangebote entscheidende Nachteile in ihrer Bildungskarriere erleben würden, die sich nachhaltig negativ auf den Wirtschaftsstandort Deutschland auswirken könnten. „Kitas und Schule zuletzt schließen und zuerst öffnen“, lautete die neue Parole der Politik. Aus kinderrechtlicher Perspektive wurden Kinder als Objekte von Bildung „entdeckt“, ohne allerdings den Subjektstatus von Kindern und den engen Zusammenhang sämtlicher Kinderrechte auf Schutz, Förderung und Teilhabe im Blick zu haben.
- Die vierte Phase erstreckte sich etwa vom Jahresbeginn bis zum Ende des Kita- und Schuljahres 2021 und erreichte ihren Höhepunkt auf dem Scheitel der dritten Welle rund um Ostern des vergangenen Jahres. Kinderärzt_innen und andere Vertreter_innen von Heilberufen hatten Alarm geschlagen, da die einschneidenden Folgen der Pandemie für die körperliche, seelische und geistige Gesundheit von Kindern immer deutlicher wurden. Im medialen und politischen Raum wurden vor allem die Ergebnisse der COPSY-Studie (Kaman et al. 2021) des Universitätsklinikums Hamburg-Eppendorf rezipiert, die deutlich machten, dass Kinder im Verlauf der Pandemie vermehrt unter psychischen Belastungen, Ängsten und depressiven Symptomen leiden.
- Phase fünf kann für den Zeitraum von Sommer bis zum Jahresende 2021 datiert werden und fällt in die Zeit des Bundestagswahlkampfes und der Bildung einer neuen Bundesregierung. Die Parteien waren sich darüber einig, dass Kitas und Schulen geöffnet bleiben sollten. Die Voraussetzungen dafür sollten u. a. durch Impfungen auch von Kindern und durch eine Verbesserung der Hygienemaßnahmen in den Einrichtungen geschaffen werden. Der Koalitionsvertrag der amtierenden Bundesregierung enthält zahlreiche Versprechungen von Vorhaben mit Bezug zu Kindern, darunter die Einführung einer Kindergrundsicherung, das Programm „Startchancen“ zur Verbesserung der Bildungschancen der Kinder unabhängig von der sozialen Lage ihrer Eltern, Verbesserungen des Kinderschutzes und einen „Nationalen Aktionsplan für Kinder- und Jugendbeteiligung“ (vgl. Koalitionsvertrag 2021, S. 93 ff.).
- Schließlich wartet eine Phase sechs weiterhin auf Verwirklichung und muss daher mit einem Fragezeichen versehen werden. Hierbei geht es um die Anerkennung und pandemiegerechte Verwirklichung sämtlicher Schutz‑, Förder- und Teilhaberechte der Kinder und des Vorrangs des Kindeswohls. Ein bedeutsames Zeichen in diese Richtung ist die Absicht der Bundesregierung, die Kinderrechte ausdrücklich im Grundgesetz zu verankern.

## Belastungen von Kindern während der Pandemie

In den (bisher) zwei Jahren der Corona-Pandemie leiden Kinder unter vielfältigen Belastungen, die sich teilweise wechselseitig verstärken. Dabei ist zu berücksichtigen, dass Kinder aufgrund ihres im Vergleich zu Erwachsenen anderen Zeitempfindens diese Jahre als besonders langdauernd wahrgenommen haben. So hat beispielsweise ein vierjähriges Kind die Hälfte seines bisherigen Lebens unter Corona-Bedingungen verbracht. Bewusste Erinnerungen an eine Zeit vor Corona sind bei diesem Kind altersbedingt nicht vorhanden.

Besonders hart belastet sind Kinder, die unter ungünstigen Lebensumständen aufwachsen. Hierzu gehören unter anderem Kinder aus Familien, die in Armut und/oder beengten Wohnverhältnissen ohne Zugang zur Natur leben, Kinder mit einem körperlich oder psychisch erkrankten Familienmitglied, Kinder mit Behinderungen oder chronischen Erkrankungen sowie geflüchtete Kinder, besonders wenn sie in Aufnahmeeinrichtungen oder Gemeinschaftsunterkünften leben (vgl. Deutsche Liga für das Kind 2020; Netzwerk Kinderrechte 2021). Im Folgenden werden zehn Belastungen beschrieben, unter denen viele Kinder gelitten haben oder weiterhin leiden:

1. *Erkrankung von Familienmitgliedern oder Bekannten:* Wenn Mitglieder der nahen oder weiteren Familie oder Freund_innen und Bekannte an COVID-19 erkranken, sind Kinder als Angehörige mit betroffen. Die Konzentration der Aufmerksamkeit auf die erkrankte Person verhindert jedoch häufig, dass Kinder in einer solchen Situation in ihrem Leid erkannt werden. Aufgrund ihres feinen Sensoriums machen sich Kinder auch dann Sorgen um eine erkrankte Person, wenn mit ihnen darüber nicht gesprochen wird. Außerdem können bei Kindern Schuldgefühle entstehen, für die Erkrankung mitverantwortlich zu sein, auch wenn diese Vorstellung einer Realitätsprüfung nicht standhält.
2. *Angst vor Ansteckung und Angst, andere anzustecken:* Schon bereits sehr junge Kinder nehmen die erwachsenen Diskurse über die Gefährlichkeit des Virus und die Risiken einer Ansteckung und Weiterverbreitung wahr und integrieren diese Befürchtungen in ihr Bild von sich selbst und anderen. Es ist immer wieder erstaunlich, wie streng sich Kinder an die Hygienebestimmungen halten und darauf bedacht sind, andere – vor allem ältere Menschen – vor einer Ansteckung durch sie bestmöglich zu schützen. Um hier keine Schuld auf sich zu laden, sind viele Kinder bereit, enorme Einschränkungen in ihrem Alltag hinzunehmen, manchmal bis zur Selbstverleugnung.
3. *Distanz von Angehörigen und Freunden:* Für die Identitätsentwicklung von Kindern ist die Verwurzelung in einem familiären und nachbarschaftlichen Netzwerk von hoher Bedeutung. Außerdem brauchen Kinder für eine gesunde Entwicklung den alltäglichen Kontakt zu anderen Kindern. Das Bedürfnis nach sozialem Austausch war jedoch aufgrund Pandemie-bedingter Restriktionen gerade für Kinder nur sehr eingeschränkt erfüllt. Während bestimmter (Lockdown‑)Zeiten war der Kontakt z. B. zu den Großeltern und anderen Angehörigen kaum oder sogar gar nicht vorhanden. Besonders belastet durch Kontaktabbrüche und Kontaktbeschränkungen waren Kinder im Falle angeordneter Quarantäne und in den Fällen, in denen altersbedingt ein Austausch über digitale Kommunikationswege nicht möglich war.
4. *Einschränkung von Bildungsgelegenheiten:* Die vorübergehenden (Teil‑)Schließungen von Kitas und Schulen haben dazu geführt, dass die Schere zwischen bildungsbevorzugten und -benachteiligten Kindern weiter auseinandergegangen ist. Wenn Eltern in der Lage waren, sich mit ihren Kindern zu beschäftigen, diese bei Bildungsanforderungen zu unterstützen und ihnen Zugang zur digitalen (Bildungs‑)Infrastruktur zu verschaffen, waren Kinder deutlich weniger belastet als Kinder in Familien, die nur über wenige Bildungsressourcen verfügen und deren Möglichkeiten der Inanspruchnahme von Unterstützung aufgrund der Pandemie stark eingeschränkt waren (Andresen et al. 2021; Langmeyer et al. 2021).
5. *Beschränkung von Spiel- und Freizeitmöglichkeiten:* Kinder haben nicht nur ein Recht auf Bildung, sondern ebenso ein Recht auf Spiel, Freizeit, aktive Erholung und Teilnahme am kulturellen und künstlerischen Leben (Art. 31 UN-Kinderrechtskonvention). Insofern stellte die oft monatelange Schließung von Sportvereinen und Schwimmbädern, Bibliotheken, Theatern, Musikschulen und zeitweise sogar Spielplätzen eine gravierende Belastung von Kindern dar, die auch nur schlecht durch nachbarschaftliche Peer-Kontakte kompensiert werden konnte, da auch hierfür Kontaktbeschränkungen galten. Lediglich die Bildschirmzeiten u. a. für Spiele und Social Media sind während der Pandemie erheblich angestiegen. So hat die durchschnittliche Gaming-Zeit von Kindern an einem Werktag auf aktuell 109 min um 31 % im Vergleich zu Vor-Corona-Zeiten zugenommen (DAK 2021).
6. *Weniger Partizipationsmöglichkeiten in Kitas und Schulen*: Die coronabedingten Kontaktbeschränkungen hatten zum Teil gravierende Auswirkungen auf die Struktur- und Prozessqualität in vielen Einrichtungen der Bildung und Betreuung sowie auf die Partizipation von Kindern: Offene bzw. altersgemischte Arbeit fiel vorgegebenen Gruppenaufteilungen zum Opfer, die Kinder hatten dadurch keine Wahlmöglichkeiten mehr, mit wem und wo sie spielen und nur noch eingeschränkten Zugang zu bestimmten Bildungsbereichen und Funktionsräumen. Schlüsselsituationen wie Essen oder Spielen verloren an Partizipationsqualität, und es ist auch von einer Beeinträchtigung der Fachkraft-Kind-Interaktion auszugehen. Regional und in den zeitlichen Phasen verschieden wurde die Maskenpflicht gehandhabt. Insbesondere für junge Kinder ist es sehr wichtig, die gesamte Mimik von Gesprächspartnern zu sehen. Auch Kinder selbst hatten ab dem Grundschulalter in Räumen stundenlang Masken zu tragen, auch, als die Maskenpflicht etwa für das Arbeiten in Büros wieder aufgehoben wurde.
7. *Zunahme familiärer Spannungen und Konflikte: *Die vorliegenden Daten zeigen, dass während der Pandemie die Spannungen und Konflikte in den Familien und damit verbunden die körperliche, seelische und sexuelle Gewalt gegen Kinder deutlich gegenüber der Vor-Corona-Zeit zugenommen hat. Die Zahl der gewaltsam in der Familie zu Tode gekommenen Kinder ist 2020 gegenüber 2019 um mehr als 30 % (von 112 auf 152 Kinder) angestiegen. Jede Woche starben im ersten Corona-Jahr etwa drei Kinder in Deutschland aufgrund von Misshandlung, Vernachlässigung oder schwerem sexuellem Missbrauch. Die Zahl der Misshandlung von Schutzbefohlenen nahm 2020 um etwa zehn Prozent gegenüber dem Vorjahr zu, die Zahl der Fälle von sexuellem Missbrauch um knapp sieben Prozent, und dies trotz einer zu vermutenden höheren Dunkelziffer aufgrund der nur eingeschränkten Verfügbarkeit pädagogischer und psycho-sozialer Dienste und Einrichtungen (Bundeskriminalamt 2021). Auch die Anzahl der Kindeswohlgefährdungsmeldungen bei den Jugendämtern hat einen neuen Höchststand erreicht und lag 2020 bei rund 60.000 gemeldeten akuten und latenten Fällen (Statistisches Bundesamt 2021).
8. *Psychische Auffälligkeiten bei Erwachsenen und Kindern:* Weiterhin stellen die bei vielen Erwachsenen im Zusammenhang mit der Pandemie auftretenden allgemeinen Zukunfts- und Existenzängste eine erhebliche Belastung für die Kinder dar. Viele Kinder müssen erleben, dass ihre Eltern die Kontrolle über ihr Leben zu verlieren drohen, starken Stimmungsschwankungen unterliegen und sich nicht selten wegen psychischer Erkrankungen in Behandlung begeben müssen. Auch die psychischen Auffälligkeiten bei Kindern haben während der COVID-19-Pandemie deutlich zugenommen. „Während der Pandemie zeigt fast jedes dritte Kind Hinweise auf psychische Auffälligkeiten wie emotionale Probleme, Verhaltensprobleme und Hyperaktivität. Vor der Pandemie war dies nur bei etwa jedem fünften Kind der Fall“ (Kaman et al. 2021, S. 36).
9. *Veränderte Lebensgewohnheiten und Mediennutzung*: Die Corona-Pandemie hat für Kinder zu veränderten und teilweise problematischen Strukturen im Tagesablauf geführt sowie zu eingeschränkter Bewegung (im Freien). „Es gibt bei Kindern einen derart klaren Anstieg an Adipositas während der coronabedingten Lockdowns, dass wir hier von einer zweiten, einer ‚stillen Pandemie‘ sprechen. Zudem beobachten wir bei Jugendlichen eine deutliche Zunahme der Neumanifestationen von Typ-2-Diabetes“ (Weihrauch-Blüher in Deutsche Adipositas Gesellschaft 2021). Seit Beginn der Corona-Pandemie haben sich zudem die Nutzungszeiten digitaler Spiele und sozialer Medien durch Kinder stark erhöht: „Gerade für Kinder und Jugendliche mit bereits davor riskanter Mediennutzung waren die Lockdowns ein erheblicher gesundheitlicher Gefährdungsfaktor, der den Übergang in eine pathologische Mediennutzung quasi katalysiert hat“ (Fischbach in Zeit online 2021).
10. *Wirtschaftliche Einbußen in der Familie:* Millionen von Arbeitnehmer_innen waren infolge der Corona-Maßnahmen von Kurzarbeit und damit verbundenen wirtschaftlichen Einbußen betroffen. Besonders hart hat es zahlreiche kleine Selbstständige, z. B. im Bereich der Gastronomie oder im Kulturbereich, getroffen, die nicht selten in ihrer wirtschaftlichen Existenz bedroht waren. Zu den Leidtragenden gehören auch die Kinder in den betroffenen Familien, die materielle Not und sozialen Abstieg ihrer Eltern erleben mussten.

## Zur Bedeutung des Kinderrechtsansatzes in Krisenzeiten

Aufgrund ihrer besonderen Verletzlichkeit und weil sie ihre Rechte nur sehr eingeschränkt selbst einfordern können, brauchen Kinder einen besonderen Menschenrechtsschutz. Dies gilt besonders in Krisenzeiten, in denen die Verteilungskämpfe härter und die Egoismen größer sind. Damit Kinder nicht nur Rechte haben, sondern ihre Rechte auch tatsächlich genießen können, ist in den mit Kindern und für Kinder tätigen fachlichen Bereichen die Einführung des Kinderrechtsansatzes (Child Rights based Approach) geboten.

Den Kinderrechtsansatz zu verwirklichen bedeutet, sämtliche Aspekte der Arbeit mit Kindern und für Kinder – u. a. Konzepte und Programme, Gestaltung des Alltags, Angebote, Umgang mit Konflikten und Beschwerden, Zusammenarbeit mit den Eltern – an den Rechten der Kinder zu orientieren. Ziel des Kinderrechtsansatzes ist es, dass jedes Kind darauf vertrauen kann, dass seine anerkannten Rechte respektiert und umgesetzt werden.

Kennzeichnend für den Kinderrechtsansatz ist, dass nicht nur nach den Bedürfnissen, sondern gleichermaßen nach den Rechten von Kindern gefragt wird. Während Bedürfnisse subjektiv und situationsabhängig sind, handelt es sich bei den Rechten der Kinder um objektive, von einzelnen Situationen unabhängige Ansprüche. Der Kinderrechtsansatz bildet den Rahmen zur Ausrichtung des Handelns von Personen und Organisationen an den Prinzipien der UN-Kinderrechtskonvention. Damit ist er ein auf die besonderen Bedürfnisse und spezifischen Rechte von Kindern ausgerichteter Menschenrechtsansatz.

Wie jeder Menschenrechtsansatz beruht der Kinderrechtsansatz auf bestimmten Prinzipien, die sich aus dem Charakter von Menschenrechten ergeben. Vor allem vier grundlegende Prinzipien können unterschieden werden: Universalität, Unteilbarkeit, Kinder als Träger eigener Rechte sowie Erwachsene als Verantwortungsträger.

- *Das Prinzip der Universalität der Kinderrechte:* Die Kinderrechte gelten weltweit in gleicher Weise für alle Kinder, unabhängig davon, in welcher Kultur oder Tradition sie leben, unabhängig auch davon, unter welchen Lebensumständen die Kinder aufwachsen. Alle Kinder sind hinsichtlich ihrer Rechte gleich. Jungen und Mädchen haben gleiche Rechte. Nicht-Diskriminierung gehört zum Kernbestand der Menschen- und Kinderrechte.
- *Das Prinzip der Unteilbarkeit der Kinderrechte:* Alle Rechte, die Kindern zustehen, sind gleich wichtig und untrennbar miteinander verbunden. Das „Gebäude der Kinderrechte“ ist als ganzheitliche Einheit zu verstehen. Keine Gruppe von Rechten ist wichtiger als eine andere. Schutz‑, Förder- und Beteiligungsrechte können gleiche Geltung beanspruchen. So sind Kinder beispielsweise besser vor Gefahren geschützt, wenn sie ihre Rechte kennen und an den sie betreffenden Entscheidungen beteiligt sind. Umgekehrt ist Partizipation auf ausreichenden Schutz angewiesen. Kinder haben das Recht sich zu beteiligen, sind dazu aber nicht verpflichtet und müssen davor geschützt werden, zur Beteiligung gedrängt zu werden.
- *Das Prinzip der Kinder als Träger eigener Rechte: *Kinder sind Träger eigener Rechte. Diese Rechte müssen von ihnen nicht erworben oder verdient werden, sie sind an kein (Mindest‑) Alter oder an kognitive oder sonstige Voraussetzungen geknüpft, die von Kindern zu erfüllen wären, und sie können von ihnen auch nicht abgelegt oder veräußert werden. Sie stehen ihnen allein deshalb zu, weil sie Kind sind, und zwar spätestens von Geburt an.
- *Das Prinzip der Erwachsenen als Verantwortungsträger:* Mit dem Prinzip der Kinder als Träger eigener Rechte korrespondiert die Pflicht der Erwachsenen, Verantwortung für die Umsetzung der Kinderrechte zu übernehmen. Erwachsene sind Pflichtenträger, von denen die Kinder die Umsetzung ihrer Rechte erwarten können. Für das Wohl des einzelnen Kindes sind in erster Linie die Eltern verantwortlich. Aber auch Staat, Wirtschaft, Kultur, Sport und Medien, Verbände und Religionsgemeinschaften sowie die verschiedenen mit Kindern tätigen Institutionen und darüber hinaus alle in einer Gesellschaft lebenden Erwachsenen tragen Verantwortung für Kinderrechte.

## Kinderrechtspolitische Lektionen aus der Krise

Neben der fachlichen Orientierung an den Kinderrechten sind als Lehren aus der Krise rechtliche Reformen notwendig, um die Position von Kindern zu stärken. Kinderrechtspolitischer Handlungsbedarf besteht vor allem in folgender Hinsicht:

- *Kinderrechte im Grundgesetz verankern: *Die Rechte des Kindes sollten als Individualrechte ausgestattet in das Grundgesetz aufgenommen werden. Im Koalitionsvertrag der Bundesregierung heißt es hierzu: „Wir wollen die Kinderrechte ausdrücklich im Grundgesetz verankern und orientieren uns dabei maßgeblich an den Vorgaben der UN-Kinderrechtskonvention“ (Koalitionsvertrag 2021, S. 98). Das hier zum Ausdruck gebrachte kinderrechtspolitische Vorhaben markiert eine günstige Ausgangsposition. Nächstes Ziel muss nun sein, einen Formulierungsvorschlag zu entwickeln, der die Vorgaben der UN-Kinderrechtskonvention maßgeblich berücksichtigt und zugleich die Chance einer verfassungsändernden Zweidrittelmehrheit in Bundestag und Bundesrat bietet. Besonders wichtig wäre es, eine Formulierung zu erreichen, der zufolge bei allen Entscheidungen das Kindeswohl als ein vorrangiger Gesichtspunkt berücksichtigt wird und Kinder an den sie betreffenden Entscheidungen beteiligt werden. Ein solcher Schritt wäre in besonderer Weise geeignet, das allgemeine Bewusstsein für die Rechte der Kinder zu stärken und ein klares Signal an Staat und Gesellschaft zu senden, das Wohlergehen und die Verwirklichung der Rechte der Kinder als zuständigkeitsübergreifende Kernaufgabe anzusehen.
- *Monitoringsystem der Kinderrechte aufbauen: *Auf allen föderalen Ebenen (Kommunen, Länder, Bund) sollte ein Monitoringsystem einander ergänzender Institutionen etabliert werden, deren Aufgabe darin besteht, die Umsetzung der Kinderrechte zu überwachen und zu fördern, Beschwerden von Kindern und Erwachsenen über Kinderrechtsverletzungen entgegenzunehmen und zu bearbeiten sowie Vorschläge für Verbesserungen zu entwickeln. Hierzu gehören auch Ombuds- und Beschwerdestellen in den besonders relevanten Institutionen Kita und Schule, aber auch in anderen Einrichtungen der Kinder- und Jugendhilfe, des Gesundheitswesens und von Kommunen. Der im Koalitionsvertrag geplante „Nationale Aktionsplan für Kinder- und Jugendbeteiligung“ und die vorgesehene Kampagne zur Information der Kinder über ihre Rechte und Beschwerdemöglichkeiten (Koalitionsvertrag 2021, S. 98) sind zu begrüßende Bausteine dafür.
- *Bekämpfung der Kinderarmut: *Der während der COVID-19-Pandemie sich noch verschärfende Teufelskreis aus materieller Armut, Bildungsbenachteiligung und gesundheitlicher Beeinträchtigung etwa jedes fünften Kindes in Deutschland muss endlich durchbrochen werden. Die im Koalitionsvertrag vorgesehene Einführung einer Kindergrundsicherung (Koalitionsvertrag 2021, S. 100) könnte ein wichtiger Schritt auf diesem Weg sein. Ergänzend ist ein Ausbau der Infrastruktur für Kinder notwendig, von der die besonders belasteten Kinder am meisten profitieren sollten.
- *Inklusion verwirklichen: *Das Gebot der Nichtdiskriminierung gemäß Artikel 2 UN-Kinderrechtskonvention und die in Artikel 24 der UN-Behindertenrechtskonvention enthaltene Verpflichtung, ein inklusives Bildungssystem aufzubauen, müssen weitaus mehr als bisher rechtlich und tatsächlich umgesetzt werden. Hierzu gehören die sozialrechtliche Zusammenlegung von Kinder‑, Jugend- und Behindertenhilfe im Sinne der sogenannten „Inklusiven Lösung“ ebenso wie ein effektives Diversity Management mit besonderem Blick auf benachteiligte Gruppen, darunter Kinder mit Behinderungen, Kinder mit Migrationsgeschichte, sozial benachteiligte Kinder, Kinder aus sozialen oder kulturellen Minderheiten und Flüchtlingskinder. Geprüft werden sollte auch, ob das vielgliedrige Schulsystem in Deutschland und die damit verbundene Tendenz zu früher Selektion, die den Zusammenhang von sozialer und Bildungsbenachteiligung verstärkt, den Anforderungen an ein inklusives Bildungssystem entspricht.
- *Wahlaltersgrenze herabsetzen: *Schließlich sollte Kindern nach dem Prinzip „Ein Mensch – eine Stimme“ das Grundrecht der Wahl eingeräumt werden. Auf diese Weise würden die Demokratie auf eine breitere Basis gestellt und die politischen Kräfteverhältnisse zwischen den Generationen neu balanciert werden. Die Interessen junger Menschen, die im Zuge des demografischen Wandels immer geringere Chancen haben, mehrheitsfähig zu sein, könnten hierdurch ein angemessenes Gewicht bekommen.

## Footnotes

1 Gemäß Art. 1 UN-Kinderrechtskonvention ist ein Kind jeder Mensch, der das 18. Lebensjahr noch nicht vollendet hat. Wenn im Folgenden von Kindern die Rede ist, sind daher Jugendliche immer mit gemeint.
